# Biochemical characterization and bacterial diversity of *Agrocybe aegerita* during postharvest storage

**DOI:** 10.3389/fmicb.2025.1596093

**Published:** 2025-05-20

**Authors:** Cairong Yang, Jiezhi Yang, Yueju Zhou, Yi Ou, ZhiMeng Wang, Weiliang Qi, Renwei Huang, Songyue Chai, Hongling Yang, Yongfang Zhou, Songqing Liu

**Affiliations:** ^1^College of Chemistry and Life Science, Chengdu Normal University, Chengdu, Sichuan, China; ^2^Sichuan Provincial Forestry and Grassland Key Laboratory of Biodiversity Conservation and Sustainable Community Development in Giant Panda National Park, Chengdu, Sichuan, China; ^3^Neijiang Academy of Agricultural Sciences, Neijiang, Sichuan, China; ^4^College of Agriculture and Forestry, Longdong University, Qingyang, Gansu, China

**Keywords:** antioxidant enzyme activities, relative abundance of bacteria, correlation analysis, *Serratia*, WGCNA

## Abstract

*Agrocybe aegerita*, as an edible delicious mushroom, the storage time and quality affect its economic value and industrial development. In the present study, *A. aegerita* was sealed and packaged in PE self-sealing bags and stored at a storage temperature of 4°C, 90% humidity. The physiological and biochemical indexes of *A. aegerita* were measured and the bacterial community on the surface of fruit was determined. Soluble protein rapidly decreased in the first 5 days and then stabilized. Superoxide anions and malondialdehyde, showed dynamic fluctuations. Antioxidant enzymes SOD and CAT exhibited variable activities, while POD remained stable. The activity of polyphenol oxidase ascended, while the total phenolic content initially dropped and subsequently registered a marginal increase. In tandem, the ascorbic acid (VC) content underwent a persistent decrease. Cell wall related enzyme displayed distinct temporal activity patterns. Analysis of bacterial diversity showed that Proteobacteria was the dominant phylum and the genus *Serratia* was the dominant bacterial genus in the storage process of *A. aegerita*. Alpha diversity analysis showed that with the extension of storage time, the bacterial diversity on the surface of the *A. aegerita* body gradually decreased. Correlation Network Analysis showed that genera *Serratia*, *Bacteroides*, and *Sphingomona*s were the most closely related bacterial genera to other bacteria, occupying a dominant position in the entire bacterial community. WGCNA showed that Altererythrobacter and Brevibacilluswere might improve the storage quality of *A. aegerita*. WGCNA also showed that *Aeromonas hydrophila* and *Acinetobacter venetianus* might disrupt the cell wall structure of *A. aegerita*. This study shed light on the understanding of the physiological indicators changes and bacterial community diversity on the surface of *A. aegerita* fruit during storage. It also provided analysis of the correlation between physiological indicator changes and bacterial community diversity on the surface of *A. aegerita* fruit, which could provide some support for scientific storage of *A. aegerita*.

## Introduction

*Agrocybe aegerita* is an edible mushroom of the genus *Agrocybe* in the family Agariculaceae, named after its wild presence on dried oil tea trees. With a protein content of 25–30% in its dried fruiting bodies, *Agrocybe aegerita* is a nutritionally significant edible mushroom (Song et al., 2020). *A. aegerita* has a delicious taste, a crispy taste, a rich and pure aroma, and a delicate texture. It can also be processed into canned product, *A. aegerita* original sauce, *A. aegerita* concentrate, and dried mushroom products. In China, people prefer to eat fresh *A. aegerita*. However, characterized by high water content and absence of protective cuticle, the fruiting bodies of *A. aegerita* are vulnerable to microbial infection, physical damage, and improper storage conditions (Zheng et al., 2023). Simultaneously, its persistent postharvest respiration and metabolism accelerate nutrient consumption, leading to cap opening, desiccation, texture softening, and malodor formation (Qu et al., 2022). Postharvest *A. aegerita* is susceptible to quality deterioration caused by endogenous factors and external environmental conditions, resulting in significantly reduced shelf life that severely compromises its commercial value and substantially impedes the sustainable development of its industry. While *A. aegerita* research emphasized cultivation (Zeng et al., 2017), bioactive compounds (Petrović et al., 2024) and preservation (Xu, 2011), studies on storage-related spoilage microorganisms are still limited.

Edible fungi is an important source of food for people. At present, there were some researches on the physiological changes of edible fungi during storage and the changes of mushroom body microorganisms. Under normal growth conditions, the production and removal of free radicals and reactive oxygen species (ROS) in cells is dynamic equilibrium, but this balance can be disrupted by storage processes (Gill and Tuteja, 2010). An antioxidant enzyme system composed of superoxide dismutase (SOD), peroxidase (POD) and catalase (CAT) is effective in eliminating ROS (Duan et al., 2011). During post-harvest storage of edible fungi, the changes of cell wall structure and components directly promoted cell separation, resulting in loose tissue structure, lower hardness and lower storage quality (Qi et al., 2015). Low temperature storage decreased the contents of electrolyte leakage, malondialdehyde (MDA), mitochondrial permeability transition pore (MPTP), Ca^2+^ and H_2_O_2_, and prolonged the shelf life of *Lentinula edodes* (Xia et al., 2024). An et al. (2021) investigated the changes in antioxidant activity, nitrite scavenging activity, and *β*-glucan content of edible mushrooms (*Pleurotus eryngii*, *Pleurotus ostreatus*, shiitake mushrooms, and *Flammulina velutipes*) stored at room temperature (20–25°C) and low temperature (4°C). They found that the iron reduction antioxidant capacity and reduction ability of air dried and baked samples stored at room temperature and low temperature show an increasing trend compared to before storage. In addition, compared with samples stored before and at low temperatures, the *β*-glucan content in air dried and baked samples stored at room temperature was significantly reduced. Scientists have also attempted to develop new methods for storing edible mushrooms. Mohebbi et al. (2012) found that the combination of *Aloe vera* and gum tragacanth was more effective to delay the decrease of storage quality of button mushroom (*Agaricus bisporus*). They discovered that gum-kefiran (GKM 0) and guar gum-kefiran-MKBE 20% (GKM 20) treatments significantly reduced the weight loss, cap structure, and delayed opening of mushrooms (Patil et al., 2024). Ultrasound, MAP with high oxygen concentration (such as 80% oxidation rate) could induce higher antioxidant capacity, significantly reduced post-harvest quality loss and maintain the texture of mushroom (Ni et al., 2018; Wang et al., 2011).

Activities of spoilage microorganism is also the important reason that leads to the decline of edible fungi storage quality. In terms of bacterial community composition, *Pseudomonas*, *Burkholderia*, *Lactococcus*, *Sphingomonas*, and *Stenotomonas* are the main bacterial populations, with significant differences in harvested edible mushrooms (*Lentinus edodes*, *Pleurotus ostreatus*, and *Hypsizygus marmoreus*) (Xia et al., 2024). Liu et al. (2023) found that the microbial community of fresh porcini transformed into spoilage after 8 days of cold storage, and the freshness of mushrooms decreased with the increase of volatile spoilage flavor. Xia et al. (2024) revealed that intestinal bacterial communities, namely, *Pseudomonas*, *Burkholderia*, *Lactococcus*, *Sphingobacterium*, and *Stenotrophomonas*, were the main bacterial communities, with significant differences among harvested edible fungi (*Lentinus edodes*, *Pleurotus ostreatus*, and *Hypsizygus marmoreus*). Jiang et al. (2023) reported that throughout the storage period, bacterial genera such as *Pseudomonas* and *Agrobacterium*, along with the family Flavobacteriaceae, and fungal taxa including *Ascomycetes*, *Aspergillus*, and *Mucor*, exerted the most significant influence on wild *Morchella*, with *Pseudomonas* demonstrating the most pronounced impact. *Pseudomonas tolaasii*, a key spoilage bacterial strain, played a major role in bringing about brown blotch in mushrooms (Mehni et al., 2023). The preservation quality of edible fungi could be improved by adding safe antistaling agent to inhibit microbial activity. Chitosan, glucose, and chitosan-glucose complex (CGC) treatments effectively maintained the tissue hardness of *Lentinus edodes* (shiitake mushrooms), suppressed the rise in respiration rate, and notably reduced microbial proliferation, including *Pseudomonas* spp., yeasts, and molds (Jiang et al., 2012). *Aloe vera* coating (3%) could reduce the number of mesophilic bacteria, yeasts, and molds, effectively prevent microbial growth, and maintain the sensory characteristics of the sample. This treatment might be a promising technique for preserving mushrooms and other vegetables and fruits (Moosavi-Nasab et al., 2023).

In the present study, *A. aegerita* were sealed and packaged in PE self-sealing bags and stored at a storage temperature of 4°C, 90% humidity. The physiological indexes of *A. aegerita* were measured at 0, 5, 15, and 20 days, respectively. The bacterial community was determined by high-throughput sequencing. To study the relationship between microbiota and metabolites, WGCNA was used to construct a network of co-occurrence taxa. This work could help us understand the physiological changes during *A. aegerita* postharvest storage and the relationship between microbial community change and metabolites.

## Materials and methods

### Experimental materials

Fresh *A. aegerita* harvested from farmers’ greenhouses in Shuangliu District, Chengdu, Sichuan Province, China, were used as experimental materials. *A. aegerita* with uniform color and luster and without pests and diseases with cap diameter of 20–40 mm were selected and sealed and packaged in PE self-sealing bags (0.1 mm thick, 17 cm × 24 cm), with 60 ± 5 g per bag. Higher storage humidity (90%) helps preserve edible mushrooms’ texture and freshness by minimizing transpiration-induced water loss, thus reducing weight loss (Zhang et al., 2020). Lowering the storage temperature (2–4°C) can delay the respiratory peak and metabolic activity of edible mushrooms, reduce water loss caused by transpiration, inhibit the growth of most spoilage microorganisms, and minimize nutrient depletion in the fruiting bodies (Giang et al., 2022). In the present study, the samples were stored in a controlled climate chamber at 4°C with 90% relative humidity for 20 days. Subsequently every 5 days, and three replicates were randomly selected for the determination of physiological and biochemical indexes and also for the 16S rRNA gene diversity analysis.

### The determination of browning analysis, weight loss analysis, analysis of membrane permeability, ROS levels, and lipid peroxidation

Browning analysis and weight loss analysis was according to Peng et al. (2020). Analysis of membrane permeability was described by Liu et al. (2010). Soluble protein was determined according to the method of Bradford (1976) using bovine Serum albumin as standard. Malondialdehyde (MDA) content was determined with reference to Jayakumar’s measurement (Jayakumar et al., 2006). O_2_^−^ were determined using assay kits purchased from Nanjing Jiancheng Bioengineering Institute (NJBI, Nanjing, China), following the manufacturer’s instructions.

### Determination of antioxidant enzyme activities and VC content

For each antioxidant enzyme activity measurement, accurately weigh 0.2 or 0.1 g fresh sample and add 1.8 or 0.9 mL of extraction solution to ice bath grinding. Then centrifuged at 560 g for 10 min at 4°C, the supernatant was taken for the determination of superoxide dismutase (SOD) or peroxidase (POD) activity. For the determination of catalase (CAT) activity, the grinded sample was centrifuged at 400 g at 4°C for 10 min. The grinded sample was centrifuged at 1,280 g at 4°C for 10 min, take the supernatant for the measurement of polyphenol oxidase (PPO). The activities of superoxide dismutase (SOD), catalase (CAT), peroxidase (POD), and polyphenol oxidase (PPO), were determined using assay kits also obtained from NJBI, following the manufacturer’s instructions. The determination of VC was referred to Li et al. (2012).

### Determination of cell wall metabolic enzyme activity

For each determination of cell wall metabolic enzyme activity, accurately weigh 0.1 g fresh sample. 1:9 ratio of sample and extract were added for ice bath grinding, centrifuged at 192 g for 10 min at 4°C, and the supernatant was taken for measurement activity of *β*-1, 3 glucan. For the measurement of cellulase activity, the grinded sample was centrifuged at 640 g at 4°C for 10 min and then the supernatant was collected. A total of 1,920 g centrifugation was carried out at 4°C for 20 min and the supernatant was taken for the determination of chitinase activity. Cell wall metabolic enzyme activity analysis cellulase activity assay method was carried out according to the kit instruction of NJBI. β-1, 3 glucanase activity assay and chitinase activity assay was carried out according to the manuals of enzyme kit of Sangon Biotech Co., Ltd. (Shanghai, China).

### Pearson correlation analysis of physiological indicators

The analysis result of Pearson correlation heatmap plot were generated using the R software packages “corrplot” through the CNSknowall,^1^ a comprehensive web service for biomedical data analysis and visualization.

### Statistical analyses of data on physiological and biochemical characteristics

Statistical analyses were conducted using the software package SPSS v24 (SPSS Inc., Chicago, United States). Specifically, one-way analysis of variance (ANOVA) was employed to assess the data, and the least significant difference (LSD) test at the 5% significance level (*p* ≤ 0.05) was utilized for pairwise comparisons of means. For data visualization, graphs were generated using Origin 2018 (OriginLab Corporation, San Diego, United States).

### 16S rDNA bacterial diversity analysis

#### DNA extraction

Total genomic DNA was extracted using DNA extraction Kit (MagPure Soil DNA LQ Kit, Magan) following the manufacturer’s instructions. Concentration of DNA was verified with NanoDrop2000 (Thermo Fisher Scientific, United States) and agarose gel. The genome DNA was used as template for PCR amplification with the barcoded primers and Tks Gflex DNA Polymerase (Takara). For bacterial diversity analysis, V3-V4 variable regions of 16S rRNA genes was amplified with universal primers 343 F and 798 R.

#### Library construction

Amplicon quality was visualized using gel electrophoresis, purified with AMPure XP beads (Agencourt), and amplified for another round of PCR. After purified with the AMPure XP beads again, the final amplicon was quantified using Qubit dsDNA assay kit. Equal amounts of purified amplicon were pooled for subsequent sequencing. Sequencing was performed using the Illumina NovaSeq 6000 platform, generating 250 bp paired-end reads. The sequencing was conducted by Shanghai OE Biotech Co., Ltd. (Shanghai, China).

#### Bioinformatic analysis

The library sequencing and data processing were conducted by OE biotech Co., Ltd. (Shanghai, China). Raw sequencing data were in FASTQ format. Paired-end reads were then preprocessed using Cutadapt software to detect and cut off the adapter. After trimming, paired-end reads were filtering low quality sequences, denoised, merged and detect and cut off the chimera reads using DADA2 (Callahan et al., 2016) with the default parameters of QIIME2 (Bolyen et al., 2019). At last, the software output the representative reads and the ASV abundance table. The representative read of each ASV was selected using QIIME 2 package. All representative reads were annotated and blasted against Silva database Version 138 (16 s rDNA) using q2-feature-classifier with the default parameters. The QIIME 2 software was employed to conduct *α* and *β* diversity analyses. Alpha diversity, which includes the Chao1 index and the Shannon index, was used to evaluate the α diversity of the samples. The unweighted Unifrac distance matrix, calculated using R (3.5.1), was utilized for unweighted Unifrac Principal Coordinate Analysis (PCoA) to assess the β-diversity of the samples. Differential analysis was performed using statistical methods such as ANOVA/Kruskal-Wallis/T-test/Wilcoxon based on the R package. Additionally, LEfSe (version 1.0.0) was applied to analyze differences in species abundance profiles.

### WGCNA analysis

#### Network construction

To study the relationship between microbiota and metabolites, WGCNA was used to construct a network of co-occurrence taxa. The first step of network construction was to select the appropriate weight parameter β (Family, optimal β = 6; Genus, optimal β = 6; Species, optimal β = 5), namely “Soft thresholding power.” With the help of power value curve, suitable soft threshold could be chosen. After choosing the appropriate soft threshold, the modules were clustered and the tree graph was drawn. Finally, the correlation coefficients between the modules and phenotypes were calculated, and the modules related to the significance of specific phenotypes were selected for downstream analysis.

#### Hub-microbiota

ExportNetworkToCytoscape is a function in the WGCNA package that exports a weighted co-expression network to Cytoscape for visualization. The threshold parameter of this function acted as a filter when exporting the network. The threshold parameter specified a threshold for edge weights, and only edges with weighted greater than or equal to that threshold were included in the exported network. The number of organisms studied varied at different taxonomic levels. As taxonomic levels decreased, species types increased, so different thresholds were chosen to filter the network (Family: 0.5; Genus: 0.5; Species: 0.9). Then, the topology of the network was investigated, and the microbes in each module were arranged from large to small according to the degree of module internality. The top 5% was taken as the Hub-microbiota. The Hub-microbiota network graph was plotted primarily using the igraph package of R (v4.3.3).

## Results

### Appearance and browning evaluation

**Figure 1:**
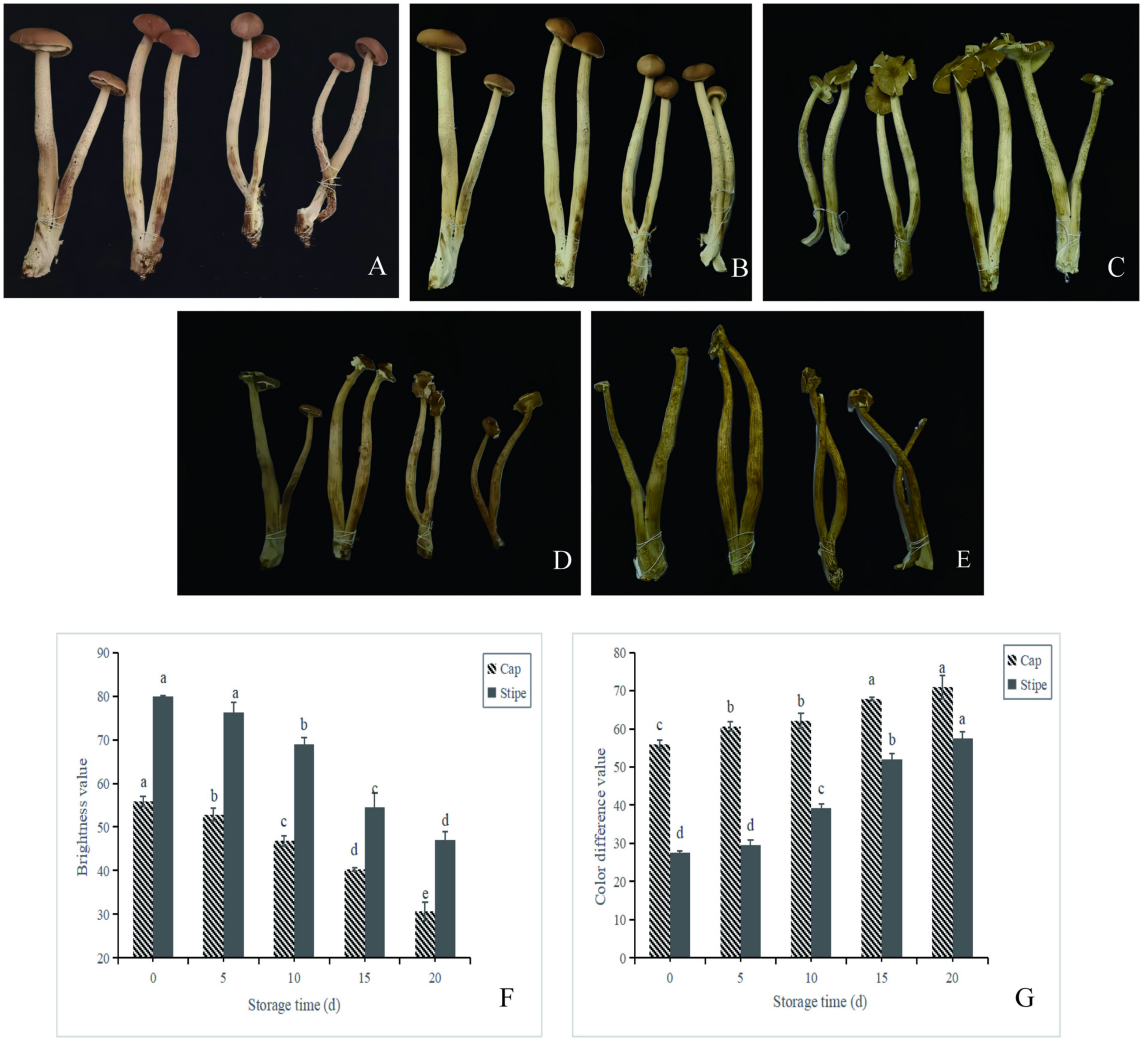
Appearance and browning evaluation of *A. aegerita* stored at 4°C and 90% RH. **(A–E)** The appearance quality of *A. aegerita* stored at 4°C and 90% RH. 0d, 5d, 10d, 15d, and 20d, respectively. **(F)** The color difference value of *A. aegerita* stored at 4°C and 90% RH. **(G)** The brightness value of *A. aegerita* stored at 4°C and 90% RH. The different lowercase letters indicate statistically significant differences (*p* < 0.05) according to Duncan’s test.

Changes in appearance, stipe, cap brightness and color difference during storage of *A. aegerita* are shown in Figure 1. With the prolongation of storage time, the browning and wilting of fruit body of *A. aegerita* body were aggravated, and the cap texture became loose and opened. The color difference of *A. aegerita* increased during postharvest storage. The color difference of the cap increased from 60 on day 0 to 71 on day 20, and the stipe increased from 28 on day 0 to 58 on day 20. The brightness values of stipe and cap showed a downward trend. The brightness of stipe decreased from 80 at day 0 to 27 at day 20, and the brightness of cap decreased from 56 at day 0 to 31 at day 20. It may be related to enzymatic browning, non-enzymatic browning, oxidation reactions or microbial activity.

### Biochemical characterization

**Figure 2:**
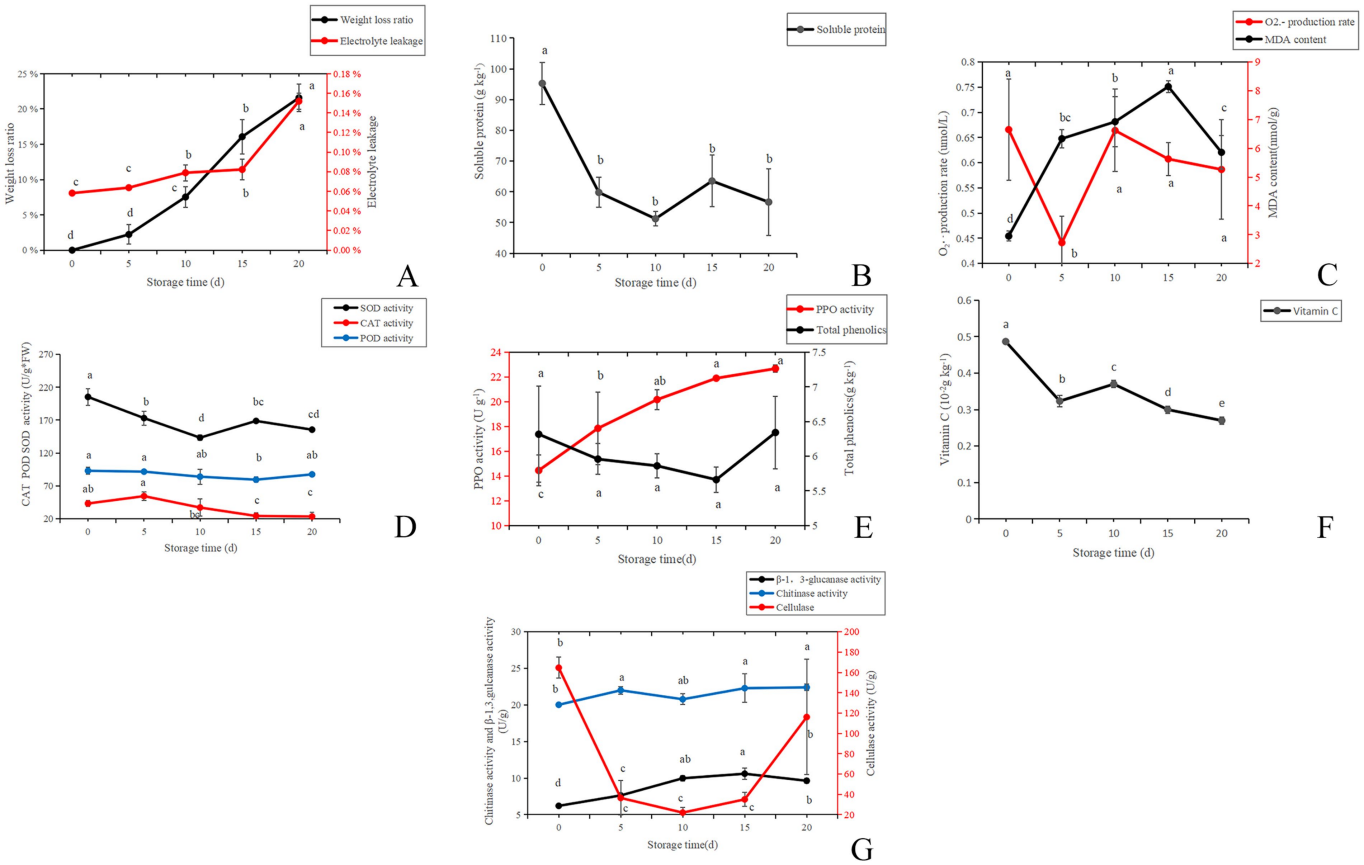
Biochemical characterization of *A. aegerita* stored at 4°C and 90% RH. **(A)** The weight loss ratio and membrane permeability of *A. aegerita* stored at 4°C and 90% RH. **(B)** The soluble protein of *A. aegerita* stored at 4°C and 90% RH. **(C)** The content of superoxide anions and MDA of *A. aegerita* stored at 4°C and 90% RH. **(D)** The SOD, CAT, and POD activity of *A. aegerita* stored at 4°C and 90% RH. **(E)** The total phenolics and PPO activity of *A. aegerita* stored at 4°C and 90% RH. **(F)** The content of VC of *A. aegerita* stored at 4°C and 90% RH. **(G)** The cell wall metabolizing enzyme activity of *A. aegerita* stored at 4°C and 90% RH. The different lowercase letters indicate statistically significant differences (*p* < 0.05) according to Duncan’s test.

With the extension of storage time, the weight loss rate of *A. aegerita* gradually increased (Figure 2A). Compared with the initial storage period (day 0), the weight loss rate on the day 15 increased by 16%, and the weight loss rate on the day 20 reached 22%. As the storage time increases, the cell permeability also gradually increased. The penetration rate increased from 6% at day 0 to 8% at day 10 and then to 15% at day 20. The results showed that the longer the *A. aegerita* was stored, the more water was lost from the fruit body, and the degree of damage to the membrane system structure also increased. Similar tendency in *C. comatus* was also reported by Peng et al. (2020).

The content of soluble protein, as a nutrient that maintains the activity of post-harvest *A. aegerita*, sharply decreased from around 95 to 60 g kg^−1^ from day 0 to day 5 (Figure 2B). After day 5, the content of soluble protein did not change significantly. By day 20, its content was around 57 g kg^−1^. At day 20, the content of soluble protein was only about 60% of the initial content.

Reactive oxygen species (ROS), for example, O_2_^•−^ and H_2_O_2_, represent oxygen metabolites with strong reactivity. They are deemed toxic by—products in the process of plant metabolism. As indicated by Desikan et al. (2004), these ROS can lead to the impairment of macromolecular substances, including lipids. During the storage process of *A. aegerita*, excessive free radicals can lead to membrane lipid peroxidation reactions, producing a large amount of membrane lipid peroxidation products. The oxidation products are mainly malondialdehyde (MDA). MDA leads to the inactivation of proteins and nucleic acids by triggering conformational changes and promoting cross—linking, which in turn results in the disruption of crucial cellular processes (Duan et al., 2011). From day 0 to day 5, the content of superoxide anions decreased from 0.67 to 0.44 μmol/L (Figure 2C). However, after 5 days, the balance of reactive oxygen species in the *A. aegerita* was severely disrupted, and the content of superoxide anions increased without insignificant change. At day 20, the content of superoxide anions increased to around 0.6 μmol/L. The oxidation product malondialdehyde increased from day 0 to 15, and then showed a decreasing trend.

The production and accumulation of reactive oxygen species during storage could cause damage to cells. SOD, CAT, and POD are antioxidant enzymes in the *A. aegerita* body and important reactive oxygen species scavengers. In the process of mushroom postharvest ripening, they played a pivotal role in antioxidant defense systems and were considered to enhance food shelf—life by protecting the integrity of cell membranes (Xing et al., 2007). The activity of SOD showed a decreasing trend from day 0 to 10, and then increased from day 10 to 15. While from day 15 to 20, SOD activity decreased again (Figure 2D). The CAT activity increased by 11 U g^−1^ from day 0 to 5, but after 5 days, its activity showed a decreasing trend, increasing the accumulation of hydrogen peroxide in the mushroom body. The change of POD enzyme activity was not significant.

Polyphenol oxidase is a type of copper containing protein that exists in the cytoplasm, cell membrane, and cell wall. According to Vámos-Vigyázo and Haard (1981), the involvement of polyphenol oxidase (PPO) in enzymatic browning was considered the primary cause of discoloration in numerous foods. During storage, PPO activity showed an upward trend, increasing from 14 U g^−1^ at day 0 to 23 U g^−1^ at day 20, which was consistent with the changes in color difference and brightness values (Figure 2E). Lo and Cheung (2005) also reported a comparable trend in *A. aegirit* var. *alba*. The total phenolic content showed a decreasing trend from day 0 to 15, followed by a slight increase thereafter. The content of VC showed a decreasing trend during the storage of *A. aegerita* (Figure 2F).

The main components of the cell wall of *A. aegerita* are chitin, *β*-1,3-glucan, and a small amount of cellulose. Buitimea et al. (2013) demonstrated that chitinase breaks down the *β*-1,4-glycoside bond in chitin, resulting in the formation of n-acetylglucosamine oligomers or monomers. Produced notably during the senescence phase of fruiting bodies, *β*-1,3-glucanase is able to break down the cell-wall *β*-glucans in *L. edodes*, which implies their participation in the autolytic process of fruiting bodies (Sakamoto et al., 2009). From day 0 to 10, the activity of β-1,3-glucanase increased, accelerating the decomposition of pectin (Figure 2G). However, the trend of enzyme activity changes in the later stage was not significant. From day 0 to 10, the activity of cellulase decreased. With the extension of storage time, the activity of cellulose gradually increased, and the decomposition of cell wall was further strengthened. The chitinase activity significantly increased from day 0 to 5, but the change was not significant thereafter.

### Correlation analysis of physiological indicators

**Figure 3:**
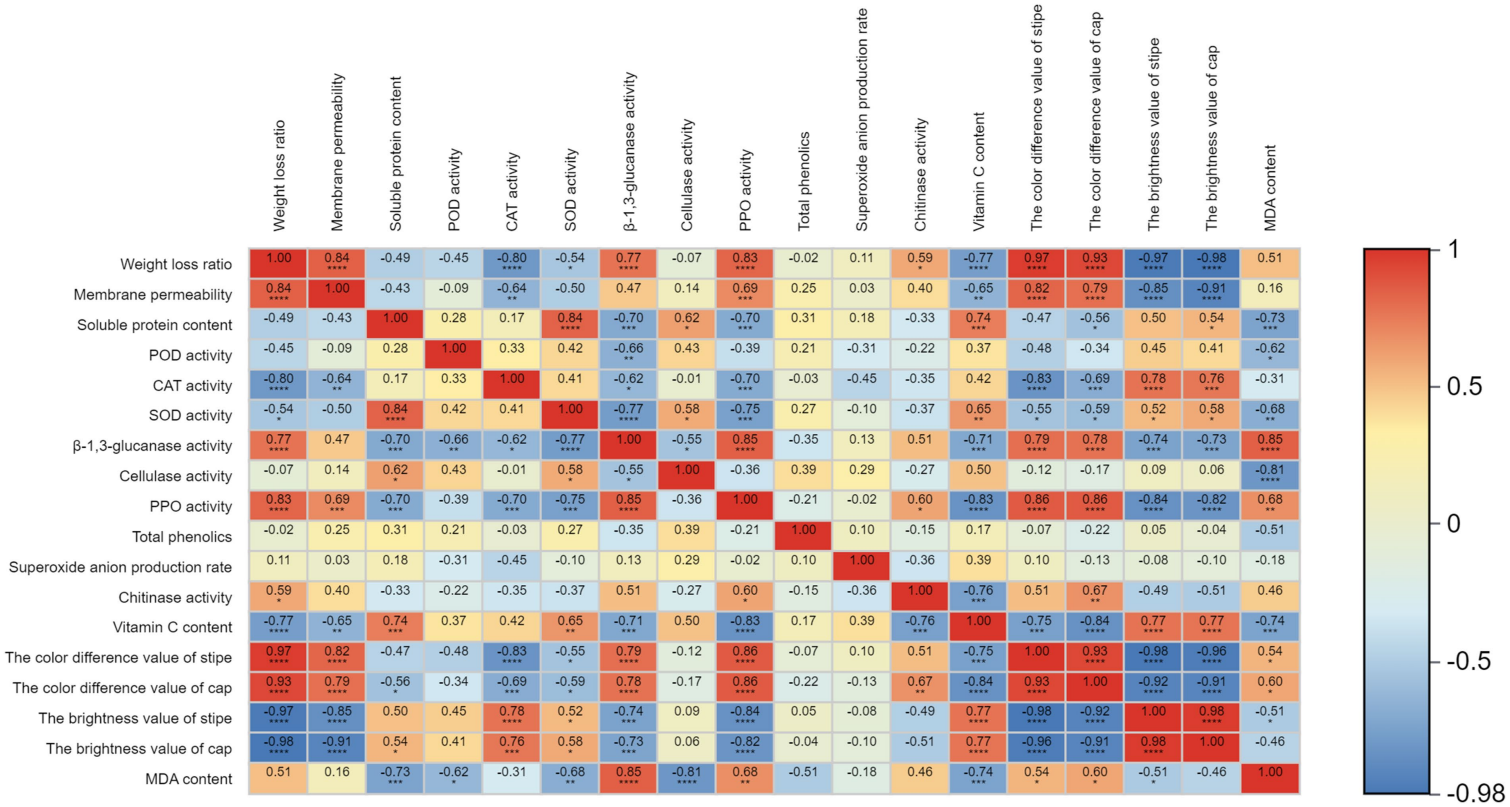
Pearson correlation analysis of physiological indicators. ****, ***, **, and * represent significant correlation at 0.0001, 0.001, 0.01, and 0.05 levels, respectively.

The results showed that CAT activity was negatively correlated with weight loss ratio (Figure 3). There was a positive correlation between PPO activity and β-1,3-glucanase, while PPO activity was negatively correlated with VC content. It showed a positive correlation between the color difference of stipe and PPO activity, β − 1, 3-glucanase. The color difference of stipe was negatively correlated with CAT activity. There was a positive correlation between the color difference of cap and PPO activity, while the color difference of cap was negatively correlated with VC content. The MDA content was positively correlated with β-1,3-glucanase and negatively correlated with the activity of cellulase.

### Analysis of bacterial diversity of *A. aegerita* during storage

**Figure 4:**
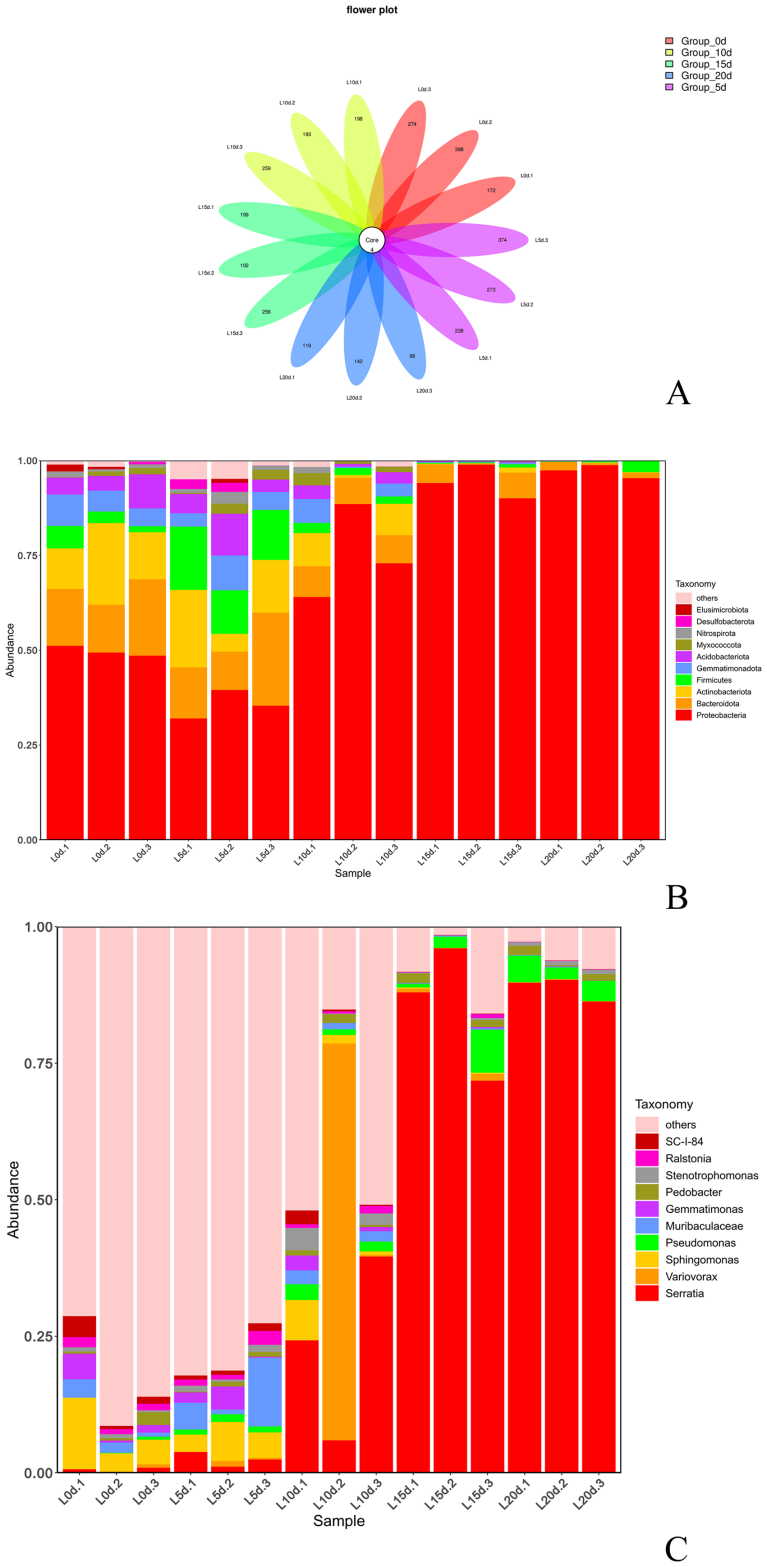
Community composition of *A. aegerita* bacteria during storage. **(A)** Venn diagram analysis of *A. aegerita* bacteria at different storage time at genus level. **(B)** Proportions of different bacteria in the samples (based on phylum level classification). **(C)** Proportions of different bacteria in the samples (based on genus level classification).

The sequencing results of samples with different storage times were clustered under 100% similarity conditions, and a total of 2,460 ASVs were obtained (Figure 4A). As shown in Figure 4A, with the extension of storage time, the number of bacterial species on the surface of *A. aegerita* fruit body slightly increased on the fifth day and then gradually decreased, showing an overall downward trend. The abundance of ASV species gradually decreased with the extension of preservation time. It was worth noting that during the storage process of *A. aegerita*, 4 ASVs belonged to genera *Serratia*, *Ralstonia*, and *Pedobacter* always existed.

As shown in Figure 4B, at the phylum level, the relative abundance of Proteobacteria is the highest during storage of *A. aegerita*, followed by Firmicutes, Actinobacteria, and Bacteroidota. The relative abundance of Proteobacteria increased with prolonged storage time, and its dominance showed an overall upward trend. The relative abundance of bacteria in the phyla Bacteroidetes, Firmicutes, and Actinobacteria decreased slightly with increasing storage time. Proteobacteria was the dominant phylum in the storage process of *A. aegerita*. At the genus level, there were significant differences in the community structure of different groups of *A. aegerita* during storage (Figure 4C). As the storage time prolongs, the types of bacteria on the surface of *A. aegerita* gradually decreased. A total of 433 bacterial genera were detected in *A. aegerita* samples, with the top four bacterial genera in relative abundance being *Serratia*, *Pseudomonas*, *Pedobacter*, and *Stenotrophomonas*. According to Figure 4C, the relative abundance of the genus *Serratia* during storage was 0.57, 2.43, 23.23, 85.27, and 88.77% from 0 to 20 days, respectively, showing an upward trend. The relative abundance of the other three genera did not change much and remained almost at the same level. Thus, the genus *Serratia* was the dominant bacterial genus during the storage process of *A. aegerita*.

**Figure 5:**
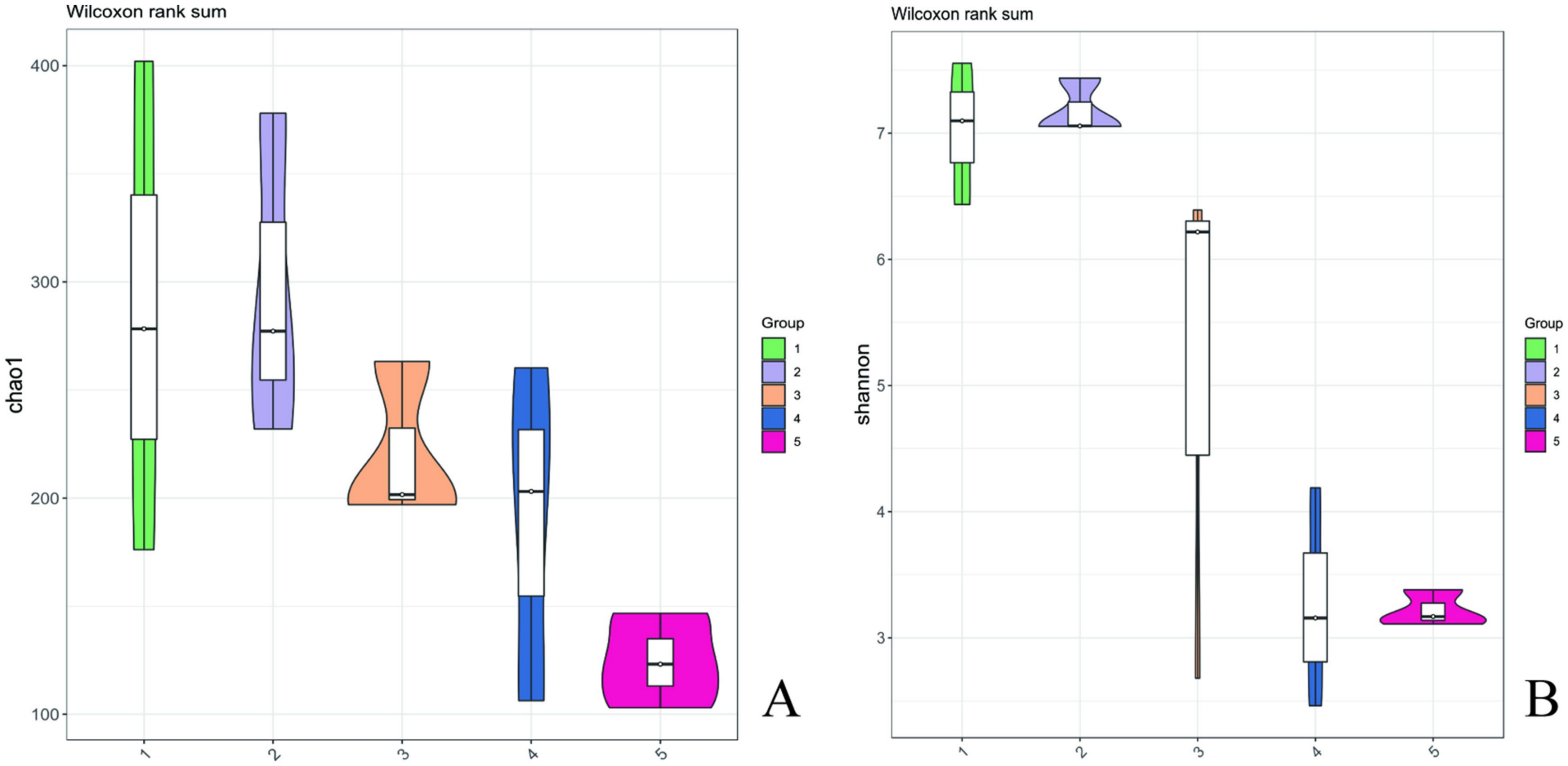
Chao1 and Shannon’s index of *A. aegerita* bacteria during storage.

As shown in Table 1, the numerical range of coverage index for each sample was greater than 0.99, indicating that almost all sequences in each sample were detected and this result could truly reflect the composition structure of bacterial diversity in the sample. Based on Table 1 and Figure 5, with the extension of storage time, both chao1 and observed species indices showed a decreasing trend. The shannon index continued to decrease, and the simpson index also showed a certain degree of decrease. During the period of day 1–5, the shannon index and chao1 index slightly increased, which might be due to the emergence of a dominant bacteria. Subsequently, the shannon index and chao1 index gradually decreased, indicating that certain bacteria gradually gained a competitive advantage and dominated. The chao1 and shannon indices of surface bacteria on *A. aegerita* stored for 15 days decreased significantly compared to day 0. The chao1 and shannon indices of *A. aegerita* bacteria stored for 20 days significantly decreased compared to day 0. This indicated that with the extension of storage time, the bacterial diversity on the surface of the *A. aegerita* body gradually decreased.

**Table 1:** Changes in alpha diversity index during the storage of *A. aegerita.*

Samples	Chao1	Shannon	Observed_species	Simpson	Goods_coverage
0d	285.4987	7.028599949	285.1333333	0.988852531	0.999945845
5d	295.74137	7.182037363	295.1333333	0.990452178	0.999928487
10d	220.608	5.095663672	220.3333333	0.803056318	0.999961119
15d	189.90027	3.269109467	189.3	0.801757107	0.999937513
20d	124.34003	3.219825272	123.7666667	0.838937439	0.999943762

**Figure 6:**
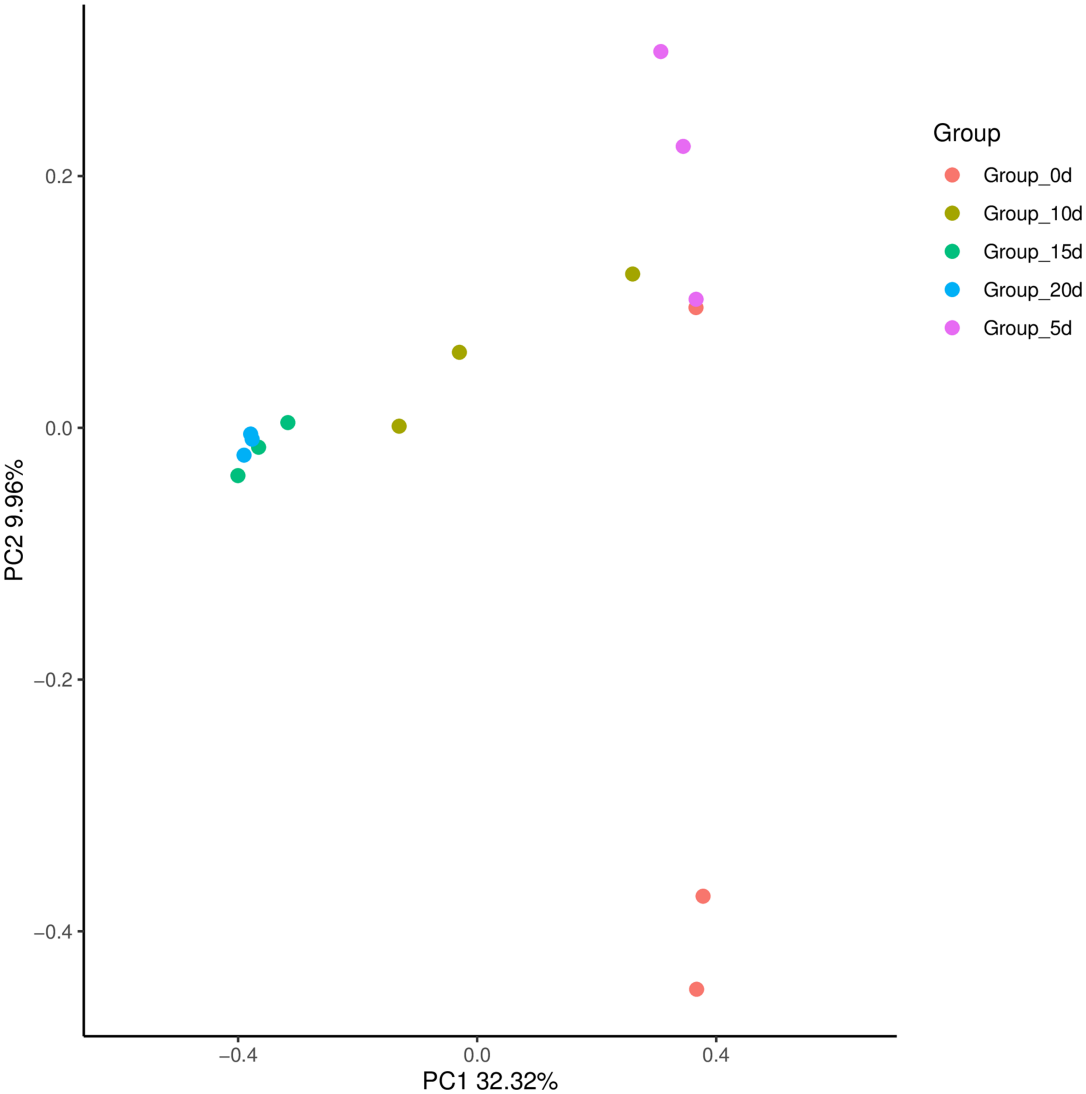
Principal coordinates of the bacterial community structure of samples.

By comparing and analyzing the species diversity of microbial communities in each group of samples, the similarity or difference in microbial community composition between each group could be explored by PCoA analysis. As shown in Figure 6, the contribution rate of the horizontal axis PC1 to the sample was 32.32%, and the contribution rate of the vertical axis PC2 was 9.96%. The samples in the same group were relatively close, while the samples in different groups were farther apart. This indicated that there were certain differences in bacterial communities between different groups of samples, and the differences in bacterial communities between samples at day 15 and day 20 were relatively small.

**Figure 7:**
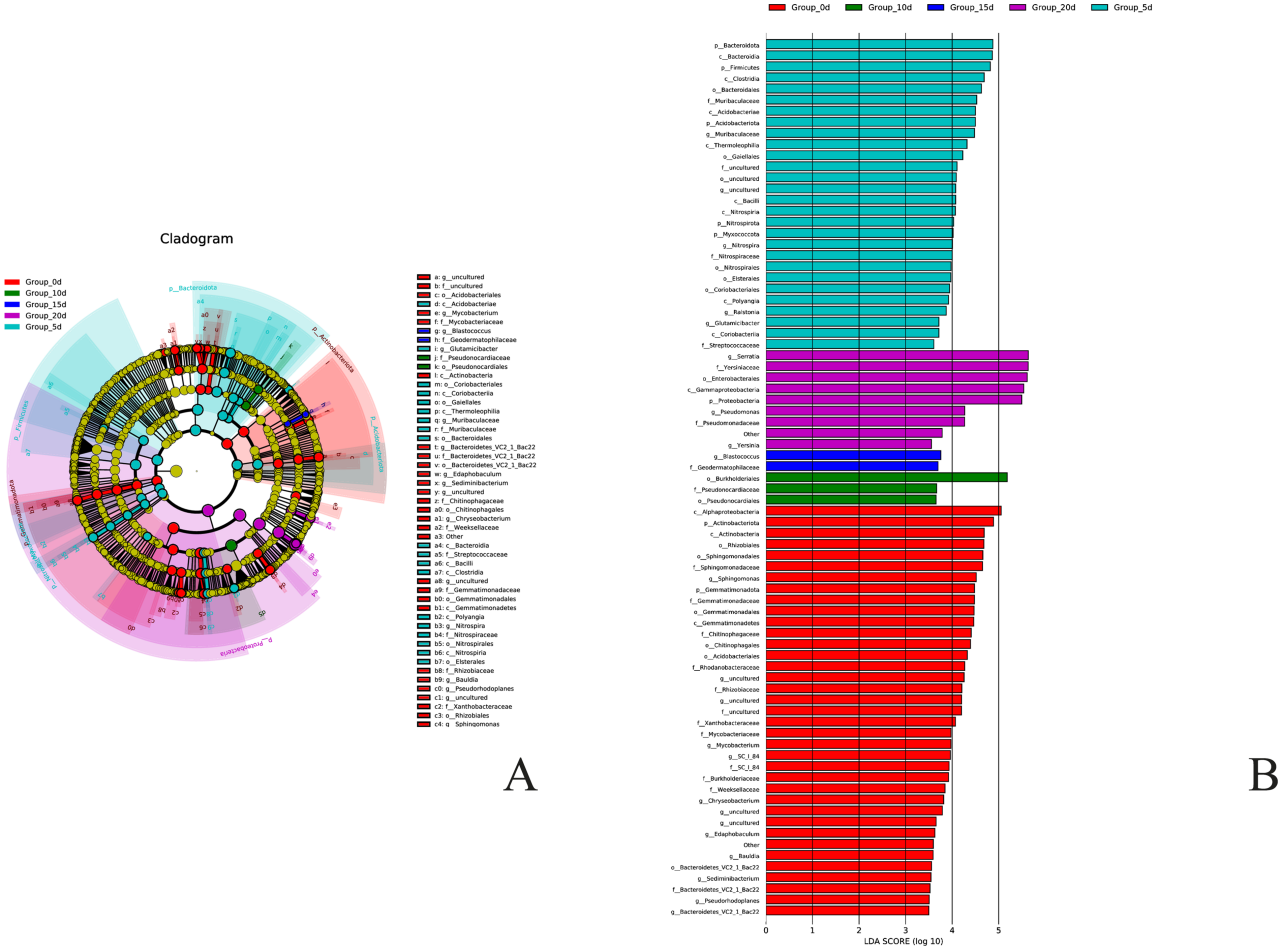
Analysis of bacteria Lefse of *A. aegerita* bacteria during storage. **(A)** Differential species annotation branching map, different colors correspond to distinct groups. **(B)** Differential species score chart. Different colors represent different groups.

Using the LEfSe analysis method to analyze the microbial community data during the storage period of *A. aegerita*, 51 diverse microbial markers were identified (screening criteria: LDA value>2.0) (Figure 7). At the phylum level, there were two bacterial phyla in the day 0 *A. aegerita* that caused significant differences in bacterial communities compared to other groups, namely Gemmatimonadota and Actinobacteria. At day 5, there were 5 bacterial phyla that caused significant differences in bacterial communities compared to other groups, namely Acidobacteria, Bacteroidetes, Myxococcata, Nitrospira, and Firmicutes. At day 10 and day 15, no significant differences were found in the core influencing bacterial phylum compared to other groups. At day 20, there was only one bacterial phylum that caused significant differences between the bacterial community and other groups, which was Proteobacteria. At the genus level, it was found that there were 9 main bacterial genera that caused significant differences in the bacterial communities of *A. aegerita* stored for 0 day compared to other groups. At day 5, there were four bacterial genera that showed significant differences between the colony and other groups, namely *Glutamicibacter*, *Muribaceae*, *Nitrospira*, and *Ralstonia*. At day 10, no bacterial genera were found that caused significant differences in bacterial communities. At day 15, there was one bacterial genus that caused significant differences in the bacterial community compared to other groups, which was *Blastococcus*. At day 20, there were two bacterial genera that caused significant differences between the bacterial community and other groups, namely *Serratia* and *Pseudomonas*, but the contribution of *Serratia* was higher than that of *Pseudomonas*. In summary, the bacterial phylum that showed significant differences compared to other storage time groups after 20 days was *Proteobacteria*, while the bacterial genera were *Serratia* and *Pseudomonas*, which were consistent with the dominant bacterial phyla and genera analyzed earlier.

**Figure 8:**
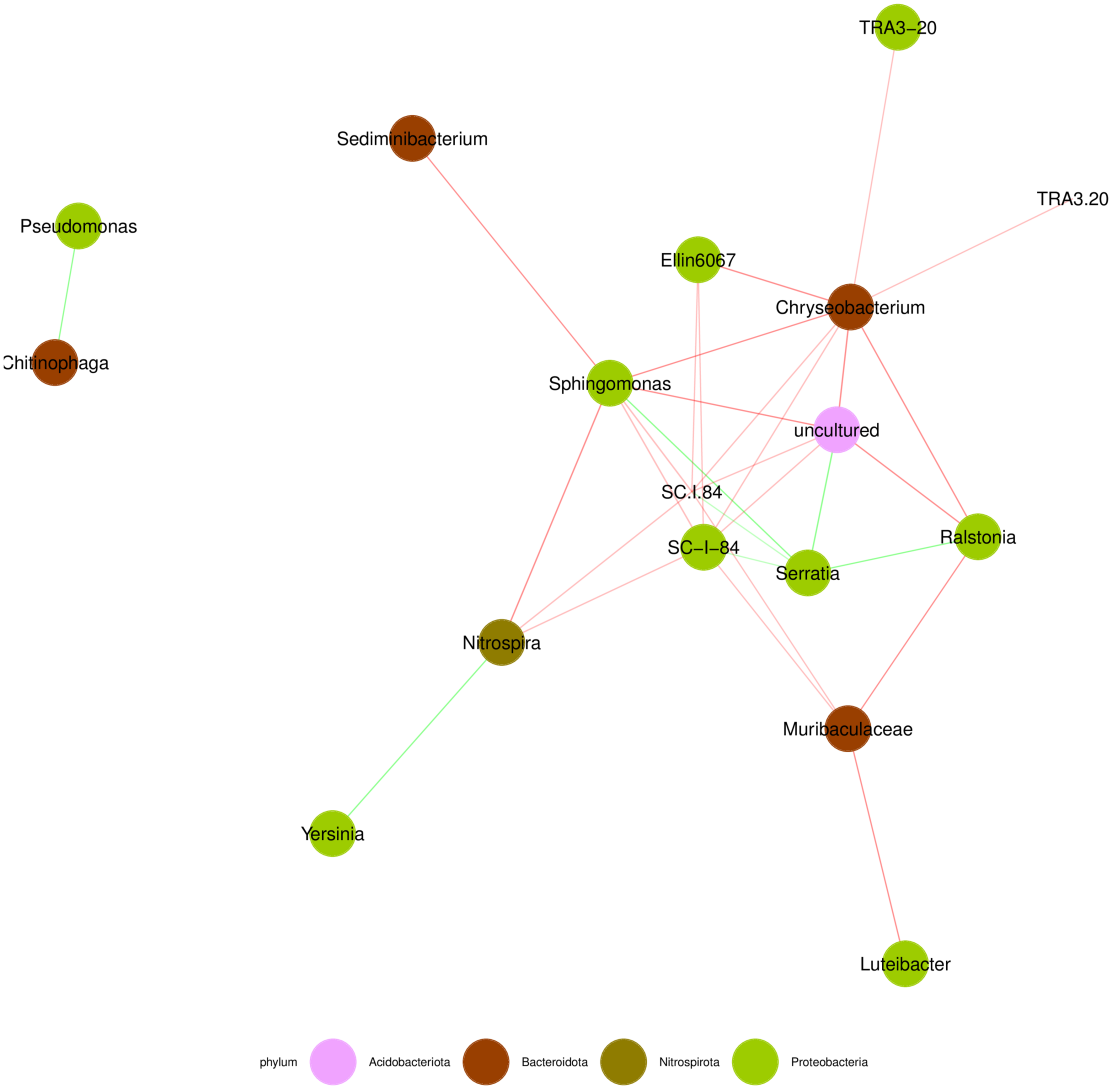
Correlation network analysis of bacteria communities at different storage time of *A. aegerita* at genus level.

The species correlation network diagram mainly reflects the species correlation at various classification levels under a certain environmental condition. Select the top 50 species in terms of total abundance at the genus level, and calculate the Spearman rank correlation coefficient between species to reflect their correlation. The default displayed in the figure shows species with |SpearmanCoef| > 0.8 and *p* < 0.01. As shown in Figure 8, the genus *Serratia* was negatively correlated with other bacterial genera, while the genus *Bacteroides* was positively correlated with other bacterial genera. The genera *Serratia*, *Bacteroides*, and *Sphingomonas* were the most closely related bacterial genera to other bacteria, occupying a dominant position in the entire bacterial community.

### Weighted gene co-expression network analysis

**Figure 9:**
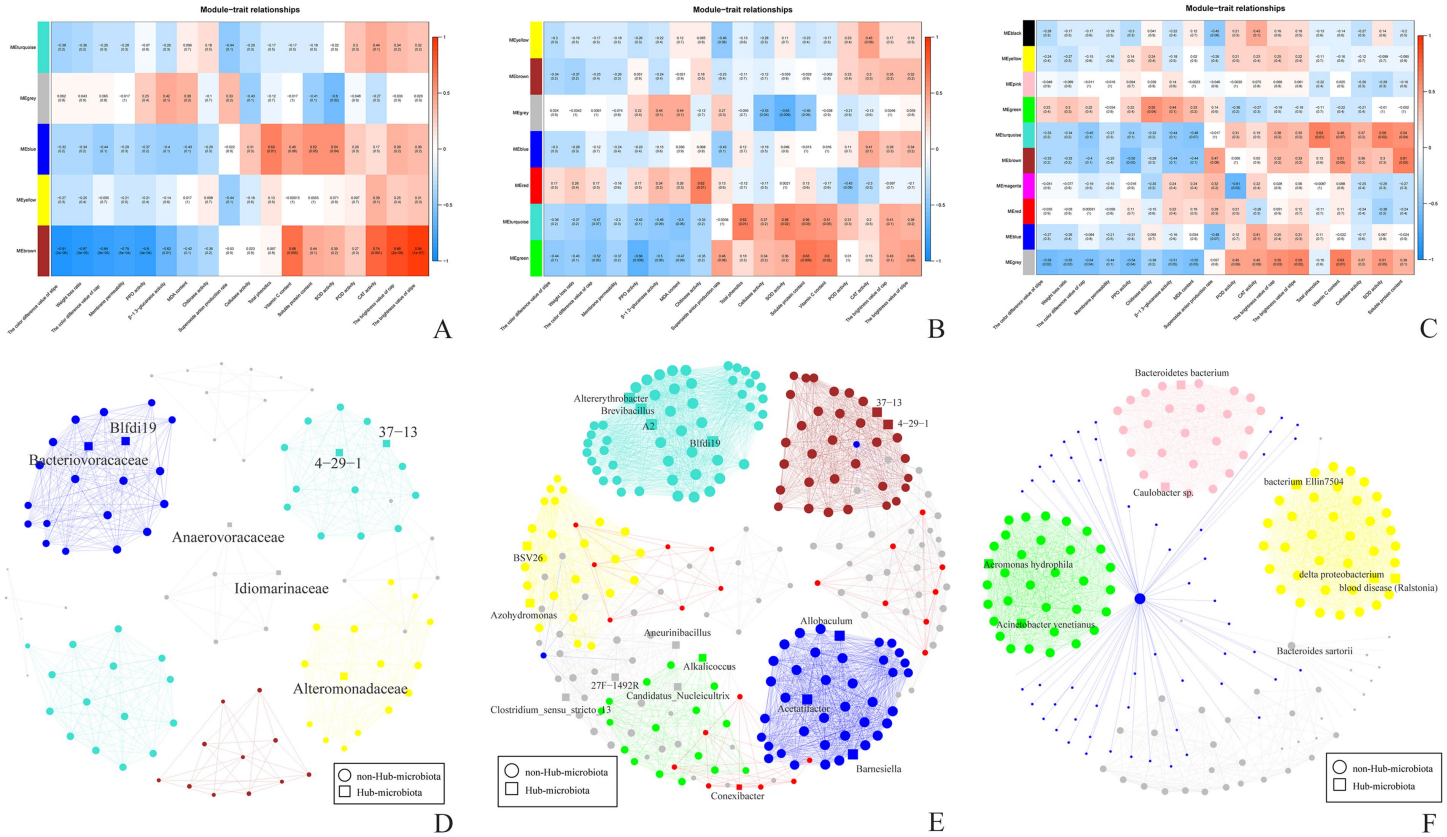
Phenotypic module correlation diagram and Hub microbiota network diagram. **(A)** Phenotypic module correlation diagram (family). **(B)** Phenotypic module correlation diagram (genus). **(C)** Phenotypic module correlation diagram (species). **(D)** Hub microbiota network diagram (Family: threshold = 0.5, hub = 5%). **(E)** Hub microbiota network diagram (Genus: threshold = 0.5, hub = 5%). **(F)** Hub microbiota network diagram (Species: threshold = 0.9, hub = 5%).

The storage quality of *A. aegerita* is strongly influenced by microbial activity. Therefore, WGCNA was conducted to detect the comprehensive correlation between the surface microbial community and physicochemical characteristics of different storage periods of *A. aegerita*. Five or seven different microbial modules (MMs) were identified at family or genus level, respectively (Figure 9). There were some interesting associations between the MMs and physicochemical characteristics. The blue module which contained family Cellulomonadaceae, Bacteriovoracaceae, Brevibacillaceae were positively correlated with total phenolics, soluble protein content and SOD activity. The VC content, CAT activity and the brightness value of cap and stipe were positively associated with family SC-1-84, Burkholderiaceae, Sphingomonadaceae and Chitinophagaceae. However, family SC-1-84, Burkholderiaceae, Sphingomonadaceae, Chitinophagaceae were negatively correlated with the color difference value of stipe and cap, weight loss ratio, membrane permeability, PPO activity and *β*-1, 3-glucanase activity. The color difference value of cap, PPO activity were negatively associated with genera *Franconibacter*, *Jatrophihabitans*, and *Alkalicoccus*. Genera *Hirschia*, *Noviherbaspirillum*, and *Conexibacter* were positively correlated with chitinase activity. Total phenolics, SOD activity, VC content and soluble protein content were positively associated with *Altererythrobacter*, *Brevibacillus*, A2 and Blfdil9. The VC content and soluble protein content were positively correlated with *Alkalicoccus*. Species *Aeromonas hydrophila* and *Acinetobacter venetianus* was positively associated with chitinase activity.

## Discussion

### Physiological and biochemical changes during the storage of *A. aegerita*

Color and texture changes are the two main factors that affect the quality and shelf life of mushrooms (Soler-Rivas et al., 1999). In our study, relatively high positive correlation (*r* ≥ 0.86) was found between the color difference and polyphenol oxidase (PPO) enzyme, which was also found in *C. comatus* after postharvest (Peng et al., 2020). It was also found that the color difference was highly positively correlated with β-1, 3-glucanase activity (*r* ≥ 0.78). In the process of storing *Lentinula edodes*, investigations revealed that glucanase activity reached its maximum value on the third day, while the activities of chitinase and cellulase demonstrated an upward trend from the third day to the sixth day (Ni et al., 2017). The modification of cell wall polysaccharides triggered by cell wall degrading enzymes was pivotal in the temperature regulated softening mechanism of postharvest *Lentinula edodes* (Li et al., 2022). Our research found that *β*-1, 3-glucanase activity increased between day 0 and 15 of storage and then decreased, while cellulase activity decreased between day 0 and 10 and then increased. The chitinase activity showed relatively small changes in the whole storage.

When subjected to biotic and abiotic stress, ROS accumulates and leads to lipid peroxidation in plants (Ren et al., 2012). The clearance of ROS by the plant’s protective system is mainly achieved through enzymatic (SOD, POD, CAT) clearance of reactive oxygen species and non-enzymatic (VC, glutathione, mannitol, and flavonoids) clearance of reactive oxygen species (Anwar et al., 2022). Superoxide anion production rate showed an upward trend during storage for day 5–10, followed by a decrease thereafter. MDA increased between day 0 and 15 and then decreased. The color difference highly negatively correlated with VC content and CAT activity. VC, as a non-enzymatic antioxidant, the content of VC gradually decreased during the storage of *A. aegerita*, gradually losing its protection for *A. aegerita*. CAT enzyme, as an important antioxidant enzyme for clearing hydrogen peroxide in the body, its activity gradually decreased after fifth day of storage, exacerbating the damage of hydrogen peroxide to *A. aegerita*. It was noticeable that the overall activity of the antioxidant enzyme system showed a downward trend, weakening the clearance of reactive oxygen species in our study. In the research of Peng et al. (2020), the activities of CAT, POD, and SOD showed an overall trend of increased first and then decreased, which to some extent protected the *C. comatus* by clearing reactive oxygen. The MDA content was highly positive correlated with β-1,3-glucanase activity. We speculated that the cell wall serves as a protective structure for the cell membrane, its destruction would exacerbate the peroxidation of membrane lipids by reactive oxygen species. Studies indicated that treatment with high carbon dioxide and low oxygen could alleviate lipid peroxidation and enhance antioxidant enzyme activity of *Pleurotus eryngii* (Liu et al., 2020). Ozone treatment showed the potential of improving storage quality of *Lentinus edodes* (Li et al., 2013). More effective and safe treatment should be developed and applied in the storage of *A. aegerita* in the future. Given that the respiration rate is a crucial parameter in storage related research, the lack of relevant data on respiration rate in this study has undeniably imposed certain constraints on our findings.

### Changes in bacterial diversity and their impact on storage quality during the storage of *A. aegerita*

Qiu et al. (2019) and Hou et al. (2023) reported that *Pseudomonas* was the predominant genus throughout the storage period of *Agaricus bisporus*. During post-harvest storage of wild morel mushrooms, *Pseudomonas*, *Pedobacter* and *Flavobacterium* were the most abundant (Jiang et al., 2023). Bacterial populations *Pseudomonas*, *Burkholderia*, *Lactococcus*, *Sphingobacterium* and *Stenotrophomonas* were predominant and varied notably in the storage of *Lentinus edodes*, *Pleurotus ostreatus*, and *Hypsizygus marmoreus* (Xia et al., 2024). In the study, as the dominant bacterial genus in the storage process of *A. aegerita*, *Serratia* continuously increased during storage, indicating that *Serratia* played an important role in the decay process of *A. aegerita* during storage. *Serratia* sp. strains exhibited the capacity to induce damage in shiitake carpophores (Tejedor-Calvo et al., 2020). Yuan et al. (2022) suggested *Serratia* may be a main spoilage bacterium causing deterioration of white *Hypsizygus marmoreus*, with correlation analysis showing a negative link to 16 volatile compounds, indicating its role in inhibiting their biosynthesis. The enlarged milk particle size and heightened acid content in milk confirmed the role of acyl-homoserine lactones (AHLs) in dairy spoilage caused by *Pseudomonas azotoformans* and *Serratia liquefaciens* (Yuan et al., 2020). The mechanistic role of *Serratia* spp. in the spoilage of edible mushrooms during storage merits further in-depth investigation in future research. Appropriate concentration of isoamyl isothiocyanate could improve storage quality by inhibiting bacterial growth, particularly the dominant bacterial community-*Serratia* on the surface of *F. velutipes* (Zhu et al., 2023). Nanocomposite-based packaging could help improve the microbial community structure of *F. filiformis* (Fang et al., 2021). During the storage of *A. aegerita*, *Serratia* should be subjected to close monitoring and controlled via suitable methodologies to mitigate its potential to induce spoilage. In accordance with the findings of Li et al. (2016), elevated temperatures and extended storage durations are expected to expedite the decline in mushroom firmness during storage. Previous study suggested that storage temperature had an impact on the concentrations of health—beneficial compounds in shiitake mushrooms (Kim et al., 2023). During the storage of *A. aegerita*, it is crucial to explore suitable environmental conditions (temperature, humidity, etc.) and storage methods.

Between the days 21 and 28 of roast chicken storage, *Pseudomonas* gradually became the main spoilage bacterium, but its relative abundance in MAP was much lower than that in normal packaging, followed by *Lachnospiraceae* and *Altererythrobacter* (Huang et al., 2020; Huang et al., 2020). However, our study discovered that *Altererythrobacter* were positively associated with total phenolics, SOD activity, VC content and soluble protein content. Wang et al. (2019) reported that *Altererythrobacter* sp. S1-5 produced acyl-homoserine lactones (AHLs) degrading enzymes that exhibit multi-target inhibitory effects against three typical aquatic pathogens (*Aeromonas hydrophila*, *Pseudomonas aeruginosa*, and *Vibrio alginolyticus*). The specific species within the *Altererythrobacter* genus responsible for beneficial effect on storage quality, along with their underlying mechanisms, required further investigation. AS spore-forming bacilli (SFB), it was reported that *Brevibacillus reuszeri* could produce enzymes that can cause spoilage of foods (Lee et al., 2016). The enrichment of milk contaminated samples at 55 degrees indicated the accumulation of mainly *Brevibacillus* and *Bacillus* (Zhao et al., 2013). Nevertheless, species of the genus *Brevibacillus* have served as an important source of antimicrobial peptides for decades, producing bioactive compounds with antibacterial, antifungal, and anti-invertebrate activities (Yang and Yousef, 2018). Liu et al. (2022) found that *Brevilaterin* B, a natural antimicrobial lipopeptide produced by *Brevibacillus laterosporus* S62-9, showed broad-spectrum antifungal activity against 33 pathogenic fungi. It was also reported that *Bacteriocin* like inhibitory substance of *Brevibacillus borstelensis* which was isolated from Marcha—a herbal cake had potential value and broad prospect in feed additives (Sharma et al., 2014). The bacteriocin-producing strain *Brevibacillus laterosporus* exhibits potent antagonistic activity against foodborne pathogens, including *Listeria monocytogenes*, *Staphylococcus aureus*, and *Clostridium perfringens* (Sharma et al., 2023). According to the WGCNA analysis, *Brevibacillus* might help improve the storage quality of *A. aegerita* by increasing total phenolics, SOD activity, VC content and soluble protein content. Future studies should both characterize the unidentified *Brevibacillus* species and elucidate whether they share conserved action mechanisms with known strains. *Aeromonas hydrophila* or *Acinetobacter venetianus* was a pathogenic and spoilage bacterium commonly found in aquatic products or rotten vegetables, proven to cause foodborne illnesses and food spoilage (Singh and Sahareen, 2017; Huang et al., 2020; Huang et al., 2020; Wang et al., 2023). The results of this study indicated that *Aeromonas hydrophila* and *Acinetobacter venetianus* might disrupt the cell wall structure of *A. aegerita* by enhancing chitinase activity.

## Conclusion

In summary, physiological and biochemical indicators of *A. aegerita* underwent changes during storage. Proteobacteria was the dominant phylum in the storage process of *A. aegerita* and the genus *Serratia* was the dominant bacterial genus during the storage process of *A. aegerita*. With the extension of storage time, the bacterial diversity on the surface of the *A. aegerita* body gradually decreased. WGCNA showed that Altererythrobacter and Brevibacillus might potentially enhance the storage quality of *A. aegerita* by increasing total phenolics, SOD activity, VC content and soluble protein content. It also showed that *Aeromonas hydrophila* and *Acinetobacter venetianus* might disrupt the cell wall structure of *A. aegerita* by enhancing chitinase activity. More attention should be paid to bacterial genera that were highly correlated with physiological changes, which might be helpful for the development of *A. aegerita* storage technology.

## Data Availability

The original contributions presented in the study are publicly available. This data can be found here https://dataview.ncbi.nlm.nih.gov/object/PRJNA1259597?reviewer=9mgqganllv7okp0oapqko7ko4p.

## Footnotes

^1^ https://cnsknowall.com/index.html#/HomePage
